# *Tabernaemontana divaricata* leaves extract exacerbate burying behavior in mice

**Published:** 2015

**Authors:** Raj Chanchal, Arumugam Balasubramaniam, Raj Navin, Sayyed Nadeem

**Affiliations:** 1 *Technocrats Institute of Technology Pharmacy, Anand Nagar, Bhopal- 462 021 (M.P.), India*

**Keywords:** *Burying behavior*, *Fluoxetine*, *Tabernaemontana divaricata*

## Abstract

**Objective:**

*Tabernaemontana divaricata* (TD) from Apocynaceae family offers the traditional folklore medicinal benefits such as an anti-epileptic, anti-mania, brain tonic, and anti-oxidant. The aim of the present study was to evaluate the effect of ethanolic extract of TD leaves on burying behavior in mice.

**Materials and Methods:**

Mice were treated with oral administration (p.o.) of ethanolic extract of TD (100, 200, and 300 mg/kg). Fluoxetine (FLX, a selective serotonin reuptake inhibitor) was used as a reference drug. Obsessive-compulsive behavior was evaluated using marble-burying apparatus.

**Results:**

TD at doses of 100, 200, and 300 mg/kg dose-dependently inhibited the obsessive and compulsive behavior. The similar results were obtained from 5, 10, and 20 mg/kg of FLX**.** TD and FLX did not affect motor activity.

**Conclusion:**

The results indicated that TD and FLX produced similar inhibitory effects on marble-burying behavior.

## Introduction

Obsessive compulsive disorder (OCD) is classified as an anxiety disorder characterized by persistent thoughts (obsessions), which are ego-dystonic and associated with seemingly purposeful behaviors (compulsions) (Goodman, 1999 ▶; Gomes et al., 2011 ▶). 

Alterations in the serotonergic system have been implicated in OCD, although the precise mechanisms underlying these abnormalities have not been identified. Only potent serotonin reuptake inhibitors (SSRIs) are consistently effective in OCD patients (Mansari and Blier, 2006 ▶). It has been reported that marble-burying behavior is inhibited by anxiolytic several selective serotonin reuptake inhibitors (SSRIs), fluvoxamine, sertraline, and paroxetine (Hirano et al., 2005 ▶). It is often used to screen anti-compulsive drugs due to high predictive and good face validity (Joel, 2006 ▶). In laboratory conditions, rats and mice spontaneously use the available bedding material to bury unpleasant sources of discomfort present in their home environment (Archer et al., 1987 ▶).

However, the mouse marble-burying test has been used as a screening model for the detection of anxiolytic activity (Prajapati et al., 2011 ▶). 

*Tabernaemontana *species (family Apocynaceae) have been reported to be rich sources of various alkaloids. *Tabernaemontana divaricata* (TD) has been used in Thai traditional medicine as components of rejuvenating and neuro-tonic remedies. It is believed that these remedies can prevent forgetfulness and improve memory (Ingkaninan et al., 2003 ▶; Chattipakorn et al., 2007 ▶; Pratchayasakul et al., 2008 ▶). 

The preliminary results obtained from a Hippocratic screening suggested that the ethanolic extracts from various parts of *T. pandacaqui *(*P*.) and *T. divaricata* (L.) possess many pharmacological activities such as central nervous system (CNS) depressant, antinociceptive, skeletal muscle relaxant (Taesotikul et al.,1989a ▶), and hypotensive activity (Taesotikul et al.,1989b ▶).

These evidences point towards TD extract against the cognitive deficits in mice (Nakdook et al., 2010 ▶), but no evidence indicated the effect of TD leaves extract on marble-burying behavior in mice. Therefore, an attempt has been made to assess the effect of TD leaves extract on marble burying behavior in mice. 

## Materials and Methods

**Plant material and extraction**

The leaves of TD were collected in January, 2010, from Bhopal, M.P., India. The plant was identified and authenticated by Dr. D.V. Amla, NBRI, Lucknow, India and a voucher No.Tit/NBRI/CIF/141/2009 specimen was deposited in Department of Pharmacognosy and Phytochemistry, TIT-Pharmacy, Bhopal, India. 

The leaves were dried in shade and stored at 25 ^°^C, powdered, and passed through sieve no. 40. 

The dried powdered leaves of TD were extracted with alcohol by maceration for 5 days. After completion of the extraction, the solvent was removed by distillation and concentrated *in vaccuo* (40 ^°^C). TD extract was subjected to phytochemical screening for thedetection of various plant constituents (Khandelwal, 2006 ▶).

**Animals**

Adult albino Swiss mice were born and reared in the animal house of TIT Pharmacy, Bhopal, from the stock originally purchased from, National Institute of Nutrition, Hyderabad, India. Young healthy male mice (21–24 g) were group housed (four per cage) in opaque polypropylene cages (28×21×14 cm^3^) and maintained at 23±2 ^°^C under 12:12 h light/dark cycle (0700–1900 h) with free access to rodent chow and tap water. The animal studies were approved by the Institutional Animal Ethics Committee (Reg. no. 831/bc/04/CPCSEA), constituted for the purpose of control and supervision of experimental animals by Ministry of Environment and Forests, Government of India, New Delhi, India. Animals were naive to drug treatment and experimentation at the beginning of all studies. Each experimental group was comprised of six mice. Testing was carried out in a counterbalanced order with respect to the treatment conditions in a noise-free room.

**Chemicals**

Fluoxetine (Cadila, India) used in present study was dissolved in 0.9 % saline. 

**Acute toxicity study**

Acute toxicity study was carried out for the TD following OECD guidelines (OECD Guidelines, 2001 ▶). The extract was dissolved in distilled water in a dose of 2 g/kg body weight and orally administered to overnight-fasted, healthy mice. The animals were observed continuously for 24 h for mortality. 

**Experimental groups**

Mice were divided into seven groups as follows: group 1 received normal saline, groups 2, 3, and 4 treated with 100, 200, and 300 mg/kg of TD, respectively, and groups 5, 6, and 7 received FLX at doses of 5, 10, and 30 mg/kg, respectively. Normal saline, TD, and FLX were orally (p.o.) administered 30 min before exposure to marble-burying apparatus.

**Assessment of marble-burying behavior**

The compulsive effect was assessed by using marble-burying behavior model in mice (Umathe et al., 2012 ▶). In brief, mice were individually placed in separate plastic cages (21×38×14 cm^3^) containing 5 cm thick sawdust bedding. 

Twenty small marbles of glass (diameter ~10 mm), were arranged on the bedding evenly spaced in four rows of five. After 30 min exposure to the marbles, mice were removed, and unburied marbles were counted. A marble was considered ‘buried’ if its two-third size was covered with saw dust. The total number of marbles buried was considered as the index of obsessive–compulsive behavior.

**Assessment of motor activity**

Motor activity was assessed in a separate group of mice using actophotometer, which had a circular arena of 40 cm, equipped with three infrared beams, and photo-cells connected to digital counter. Motor activity was assessed in terms of total number of counts of light beams interruptions in 30 min (Gaikwad et al., 2007 ▶).

**Statistical analysis**

The results are expressed as mean±S.E.M. Data were analyzed using one-way analysis of variance (ANOVA) followed by Tukey’s multiple comparison test. p*<*0.05 was considered statistically significant in all the cases.

## Results

**Phytochemical screening of TD**

Phytochemical screening revealed that TD showed the presence of alkaloids, proteins, amino acids, flavonoids, saponins, phenols, glycosides, tannis, steroids, triterpenoids, fixed oils, and fats (Table 1).

**Table 1:** Phytochemical screening of *Tabernaemontana divaricata*

**Test**	**Ethanol Extract**
**Alkaloids**	+
**Carbohydrates**	-
**Proteins**	+
**Amino Acids**	+
**Flavonoids**	+
**Saponins**	+
**Phenols**	+
**Glycosides**	+
**Tannis**	+
**Steroids**	+
**Triterpenoids**	+
**Fixed Oils**	+
**Fats**	+

**Acute toxicity**

TD was found to be safe at a dose of 2000 mg/kg, p.o. Therefore, the doses of 100, 200, and 300 mg/kg, p.o. were selected for in vivo pharmacological studies. 

**Effect of TD and FLX on marble-burying behavior in mice**

**Figure 1:**
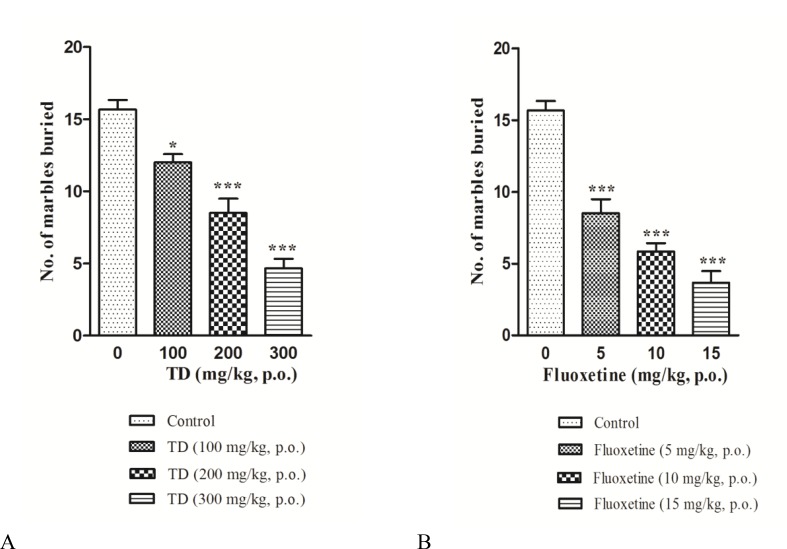
Effect of acute treatment with* Tabernaemontana divaricata* (TD) (A), fluoxetine (B) on marble-burying behavior in mice. Values are expressed as mean±S.E.M (n = 6). Values are statistically significant at ^***^p<0.001, ^*^p <0.05 vs. respective control group (One-way ANOVA followed by Tukey’s post hoc test).

One-way ANOVA followed by Tukey’s test revealed that acute treatments with 100, 200, and 300 mg/kg of TD (F=40.28, p<0.0001) and 5, 10, and 20 mg/kg of FLX (F=40.28, p<0.0001) dose-dependently reduced marble-burying behavior in mice when compared to control group (Figure 1). 

**Figure 2:**
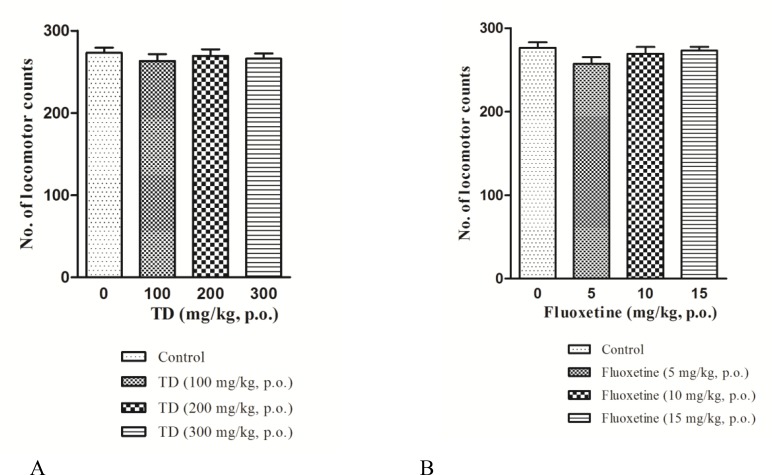
Effect of acute treatment with* Tabernaemontana divaricata *(TD) (A), fluoxetine (B) and vehicle on locomotor activity of individual mice was assessed. Values are expressed as mean±S.E.M (n = 6). p>0.05 compared to the control-treated group. (One-way ANOVA followed by Tukey’s post hoc test

Post hoc test revealed that TD at doses of 100, 200, and 300 mg/kg (F=0.3677, p=0.7771) and FLX at doses of 5, 10, and 20 mg/kg (F=0.3677, p=0.7771) did not affect motor activity when compared with normal saline group (Figure 2).

## Discussion

The result of this study shows that ethanolic extract of TD has positive effect on obsession and compulsion and generalized anxiety in mice. In addition, the results did not show any mortality and any serious side effects when the mice fed orally with TD at a dose of 2000 mg/kg.

Some investigators have opinion that the marble-burying behavior model is unable to differentiate anxiolytic and anticompulsive agents (Treit et al., 1981 ▶; Broekkamp et al., 1986 ▶). It may be because anxiolytics impair locomotor activity, and thereby affect marble-burying behavior (Pelleymounter et al., 2002 ▶; Li et al., 2006 ▶). However, in the present study, the given doses of TD or FLX did not affect locomotor activity. Serotonergic hypothesis of fear or anxiety behavior proposed that in stressonergic or threating situations the serotonergic system activity increases where as the reduction of serotonergic systems exert anxiolytic-like effects (Venkata et al., 2008 ▶). This suggestion is consistent with the observation that many OCD patients benefit from the use of SSRIs, a class of antidepressant medications that allows for more serotonin to be readily available to other nerve cells (Prajapati et al., 2011 ▶). The studies revealed that increased marble-burying behavior in control group state might be consequent to the inhibitory effect of alcoholic extract of TD and FLX. In addition, Hippocratic screening study reported that ethanolic extract of TD was found to cause dose-related decreased motor activity, ataxia, loss of righting reflex, decreased respiratory rate, and loss of screen grip. These effects indicated that TD has depressive effects on the central nervous system (Taesotikul et al., 1989 ▶).

Phytochemical screening revealed the presence of alkaloids, flavonoids, steroids, saponins, terpenes, and phenolic compounds in TD, so it may be possible that the mechanism of anti-compulsive action of TD may be due to involvement of any of these phytoconstituents in serotonergic neurotransmission. Moreover, triterpepenoids (steroidal compounds), which are able to cross blood brain barrier (BBB) due to their lipophilic nature, are present in the TD leaves. Hence, the observed effect of TD in obsessive compulsive disorder could be related to serotonergic modulation and anti-compulsive activity at a molecular level in central nervous system.

In conclusion, ethanolic extract of TD leaves and FLX, reduced obsessive and compulsive symptoms and produced anti-anxiety effects. 
